# Development and validation of a nomogram to predict complications in patients undergoing simultaneous bilateral total knee arthroplasty: A retrospective study from two centers

**DOI:** 10.3389/fsurg.2022.980477

**Published:** 2022-09-14

**Authors:** Kuishuai Xu, Liang Zhang, Zhongkai Ren, Tianrui Wang, Yingze Zhang, Xia Zhao, Tengbo Yu

**Affiliations:** ^1^ Department of Sports Medicine, the Affiliated Hospital of Qingdao University, Qingdao, China; ^2^ Department of Abdominal Ultrasound, the Affiliated Hospital of Qingdao University, Qingdao, China; ^3^ Department of Traumatology, the Affiliated Hospital of Qingdao University, Qingdao, China

**Keywords:** simultaneous bilateral, total knee arthroplasty, age, nomogram, validation

## Abstract

**Purpose:**

Complications were significantly increased 30 days after Simultaneous bilateral total knee arthroplasty (SBTKA). In this study, an individualized nomogram was established and validated to predict the complications within 30 days after SBTKA.

**Methods:**

The general data of 861 patients (training set) who received SBTKA in The Affiliated Hospital of Qingdao University between January 1, 2012 and March 31, 2017 were retrospectively analyzed. All patients were divided into complication group (*n* = 96) and non-complication group (*n* = 765) according to the incidence of complications within 30 years after SBTKA. Independent risk factors for postoperative SBTKA complications were identified and screened by binary logistic regression analyses, and then a nomogram prediction model was constructed using R software. The area under curve (AUC), calibration curve, and decision curve analysis (DCA) were selected to evaluate the line-chart. Meanwhile, 396 patients receiving SBTKA in the Third Hospital of Hebei Medical University who met the inclusion and exclusion criteria (test set) were selected to verify the nomogram.

**Results:**

Five independent predictors were identified by binary logistic regression analyses and a nomogram was established. The AUC of this nomogram curve is 0.851 (95% CI: 0.819–0.883) and 0.818 (95% CI: 0.735–0.900) in the training and testing sets, respectively. In the training set and test set, calibration curves show that nomogram prediction results are in good agreement with actual observation results, and DCA shows that nomogram prediction results have good clinical application value.

**Conclusion:**

Older age, lower preoperative hemoglobin level, higher preoperative blood urea nitrogen (BUN) level, longer operation time, ASA grade ≥ III are independent predictors of SBTKA complications within 30 days after surgery. A nomogram containing these five predictors can accurately predict the risk of complications within 30 days after SBTKA.

## Introduction

Knee osteoarthritis is the most common degenerative disease of the synovial joint, and its incidence increases with age (1). Total knee arthroplasty (TKA) is an effective method for the treatment of advanced knee diseases (2). One-third of patients have bilateral knee symptoms, and about 20% of patients need bilateral total knee replacement (3). The safety and efficacy of SBTKA versus staged bilateral TKA remains controversial in current orthopedic practice for patients requiring bilateral total knee replacement.

Studies have found that after SBTKA, the infection rate and length of hospital stay of patients are reduced, but the incidence of 90-day mortality, venous thromboembolism and neurological complications is increased (4). In addition, the risk of pulmonary embolism, cerebral embolism (5), blood transfusion rate (6, 7), cardiac complications, urinary retention, and deep infection are significantly increased (8). At present, the comparative studies after SBTKA and staged bilateral TKA are relatively common, and many studies have provided a comprehensive assessment of complication incidence, clinical efficacy and mortality after SBTKA (4, 8–10). Due to the lack of established indications for SBTKA, it is always controversial to determine the age of patients, and previous studies only confirmed that patients aged 75 and above are generally not recommended to undergo SBTKA (11–13). Recent studies by scholars (14) also only confirmed that patients under 75 years of age and ASA1 grade or grade 2 received SBTKA, and the incidence of complications was no different from that of unilateral TKA. For complications after TKA, some scholars used the nomograms prediction model to predict the probability of complications within 30 days after primary TKA (15). Since the incidence of complications after bilateral TKA increased significantly during the same period, it was necessary to establish a nomogram prediction model for complications after SBTKA and identify high-risk patients as early as possible in order to reduce the risk of postoperative complications.

The purpose of this study is as follows: a retrospective study of 861 patients from The Affiliated Hospital of Qingdao University was conducted to determine the occurrence of complications 30 days after SBTKA, establish a nomogram model to predict complications 30 days after SBTKA, and include clinical information of 396 patients from the Third Hospital of Hebei Medical University for nomogram verification.

## Methods

This study was designed as a retrospective cohort study. Patients who underwent SBTKA at the Affiliated Hospital of Qingdao University on January 1, 2012 and March 31, 2017 were enrolled in this study. Inclusion criteria: (1) Simultaneous bilateral total knee arthroplasty in the Affiliated Hospital of Qingdao University; (2) No history of knee joint infection: (3) The case data were complete, and all patients signed the informed consent. Exclusion criteria: (1) SBTKA performed at the same time as other operations; (2) Incomplete data or patients lost to follow-up. In addition, from March 2015 to September 2019, we enrolled 396 patients who underwent SBTKA at the Third Hospital of Hebei Medical University and met the inclusion and exclusion criteria to create the test set.

We determined 30-day postoperative complications and mortality using 30-day outpatient follow-up in an electronic clinical data system, as well as further information obtained from medical records and telephone follow-up. Consistent with the existing literature (16–18), we defined the 30-day postoperative complications as any of the following:Hemorrhage requiring transfusion of ≥4 U red blood cells within 72 h after surgery, delirium, pulmonary embolism, postoperative sepsis, septic shock, cerebral infarction, acute renal failure, cardiac disease (coronary artery disease, congestive heart failure, valvular insufficiency, arrhythmias, cardiac arrest requiring CPR), urinary tract infection, deep vein thrombosis, pneumonia, deep wound infection/interorgan infection, peripheral nerve injury, superficial wound infection, gastrointestinal ulcer or bleeding. Indications for blood transfusion at our institution were patients with Hb < 70 g/L or Hb < 80 g/L but symptoms of anemia. Finally, a total of 861 cases meeting the inclusion and exclusion criteria were included in this study. This study was approved by the Ethics Committee of the Affiliated Hospital of Qingdao University (Approval No.: QYFYWZLL26915).

The variables collected in our study are shown in Table 1. All the data were collected from the electronic medical record system of our hospital, and a unified and standardized survey form was designed, which was collected by two joint surgeons independently, and the controversial data was discussed and determined by the third joint surgeon and the two data collecting physicians.

**Table 1:** Univariate analysis of clinical data of the two groups.

Variable	No complications (*n *= 765)	Complications (*n* = 96)	*t*/*z*/*χ*^2^	*P*
**Gender**			1.524	0.217
Male	134 (17.52)	12 (12.5)		
Female	631 (82.48)	84 (87.5)		
Age (years)	63.54 ± 6.60	65.88 ± 6.74	−3.212	0.002*
Course of disease (year)	9.13 ± 5.68	9.89 ± 7.83	−0.916	0.362
**Priority site**			0.435	0.509
On the left side	722 (94.38)	89 (92.71)		
On the right side	43 (5.62)	7 (7.29)		
BMI (kg/m^2^)	27.62 ± 3.94	27.18 ± 4.19	1.016	0.310
Smoking	80 (10.46)	10 (10.42)	0.000	0.990
Alcohol	73 (9.54)	8 (8.33)	0.146	0.702
Operation history	274 (35.82)	41 (42.71)	1.746	0.186
Transfusion history	39 (5.10)	8 (8.33)	1.730	0.188
**ASA status**			6.680	0.010*
1 or 2	699 (91.37)	80 (83.33)		
3 or 4	66 (8.63)	16 (16.67)		
**Anesthesia**			0.037	0.848
General anesthesia	174 (22.75)	21 (21.88)		
Intraspinal anesthesia	591 (77.25)	75 (78.12)		
Duration of the operation	117.44 ± 33.98	127.02 ± 28.60	−2.647	0.008*
**Laboratory tests**				
WBC (109/L)	6.23 ± 1.87	5.73 ± 1.38	1.845	0.066
PLT (109/L)	234.15 ± 63.32	225.69 ± 65.27	0.889	0.375
Hb (g/L)	133.99 ± 10.89	125.75 ± 20.38	3.893	0.000*
Hct (%)	40.40 ± 3.79	38.47 ± 5.86	3.155	0.002*
BUN (mg/dl)	9.17 ± 6.24	16.65 ± 4.74	−14.022	0.000*

WBC, white blood cells; PLT, platele; tBMI, body mass index; Hb, hemoglobin; Hct, hematocrit; BUN, blood urea nitrogen; ASA, American society of anesthesiologists.

*P < 0.05.

All the operations are performed by experienced surgeons in our hospital. Both knees were done by the same surgeon. All patients underwent a median incision with a medial parapatellar approach. 412 patients received medial pivot prosthesis (Advance Medial-Pivot Knee System, Wright Medical Group, Arlington, TN) and 449 patients received posterior knee stabilization prosthesis (NexGen LPS-Flex, Zimmer, Warsaw, IN) without conventional patella trimming. After completing the surgery on one side, the contralateral tourniquet was pumped and the contralateral operation continued, as above. Negative pressure drainage tubes were placed in each knee joint of each patient after surgery, and all drainage tubes were removed within 24 h. TXA was injected intravenously half an hour before surgery to prevent bleeding. All patients received anti-thrombotic elastic socks combined with low molecular weight heparin for the prevention of deep vein thrombosis, and all patients received daily rehabilitation training with the help of professional therapists.

### Statistical analysis

All statistical analyses were performed with SPSS (version 24, IBM, USA) and R software (version 3.6.1, R Foundation for Statistical Computing, Austria). Measurement data were expressed as mean and standard deviation (x ± s), while classification data were expressed as quantity (percentage). Chi-square test or Fisher's exact probability method was used to analyze the differences of classification data in different age groups. The independent sample *t* test was used to analyze the differences in the measurement data between the complication group and the non-complication group, and the chi-square test or Fisher's exact probability method was used to analyze the differences in the classification data between the two groups. Subsequently, the indicators with statistical significance in univariate analysis were included in multivariate Logistic regression analysis, and the indicators with statistical significance (*P* < 0.05) were finally screened out as predictors for inclusion in the model. A nomogram was established based on the independent predictors in R software. The area under the curve(AUC) based on the receiver operating characteristic (ROC) curve was used to evaluate the discrimination of the nomogram. Furthermore, the calibration curve was used to evaluate the calibration of the nomogram, and decision curve analysis (DCA) was used to estimate the clinical usefulness of the nomogram in the training and testing sets. In addition, the ROC curves of each independent predictor were established, and comparisons of the AUCs between the nomogram and independent risk factors was performed. Test level *α *= 0. 05.

## Results

A total of 861 patients underwent SBTKA in the Affiliated Hospital of Qingdao University between January 1, 2012 and March 31, 2017, including 146 males and 715 females. The mean age of patients was 63.8 ± 6.65 years (range, 31–82 years), and the mean body mass index (BMI) was 27.53 ± 4.11 kg/m^2^ (range, 17.57–56.16 kg/m^2^).

### Independent risk factors for complications within 30 days after SBTKA in the training set

Baseline data of 765 patients in the non-complication group and 96 patients in the complication group were analyzed by univariate analysis. The results indicated that the differences in age, hemoglobin, hematocrit, urea nitrogen, operation time and ASA grade were statistically significant (*P *< 0.05), other variables had no statistical significance (*P *> 0.05) (Table 1). The occurrence of complications was taken as the dependent variable, and the significant indicators in the baseline data were included in the multivariate Logistic regression analysis. The results indicated that older age, lower preoperative hemoglobin levels, higher preoperative BUN levels, longer operation time, and ASA grade ≥ III were independent risk factors for predicting complications after SBTKA (Table 2).

**Table 2:** Multivariate logistic regression analysis of early complications after SBTKA.

	B	SE	Wald	OR	95% CI	*P*
Hb	−0.057	0.017	11.478	0.944	0.913–0.976	0.001*
Hct	0.099	0.057	2.964	1.104	0.986–1.235	0.085
BUN	0.139	0.017	67.667	1.149	1.111–1.187	0.000*
Age	0.046	0.018	6.472	1.047	1.011–1.086	0.011*
Duration of the operation	0.008	0.003	5.257	1.008	1.001–1.014	0.022*
ASA	−1.309	0.368	12.689	0.270	0.131–0.555	0.000*

Hb, hemoglobin; Hct, hematocrit; BUN, blood urea nitrogen; ASA, American society of anesthesiologists.

*P < 0.05.

### Development of a nomogram to predict complications within 30 days after SBTKA

**Figure 1:**
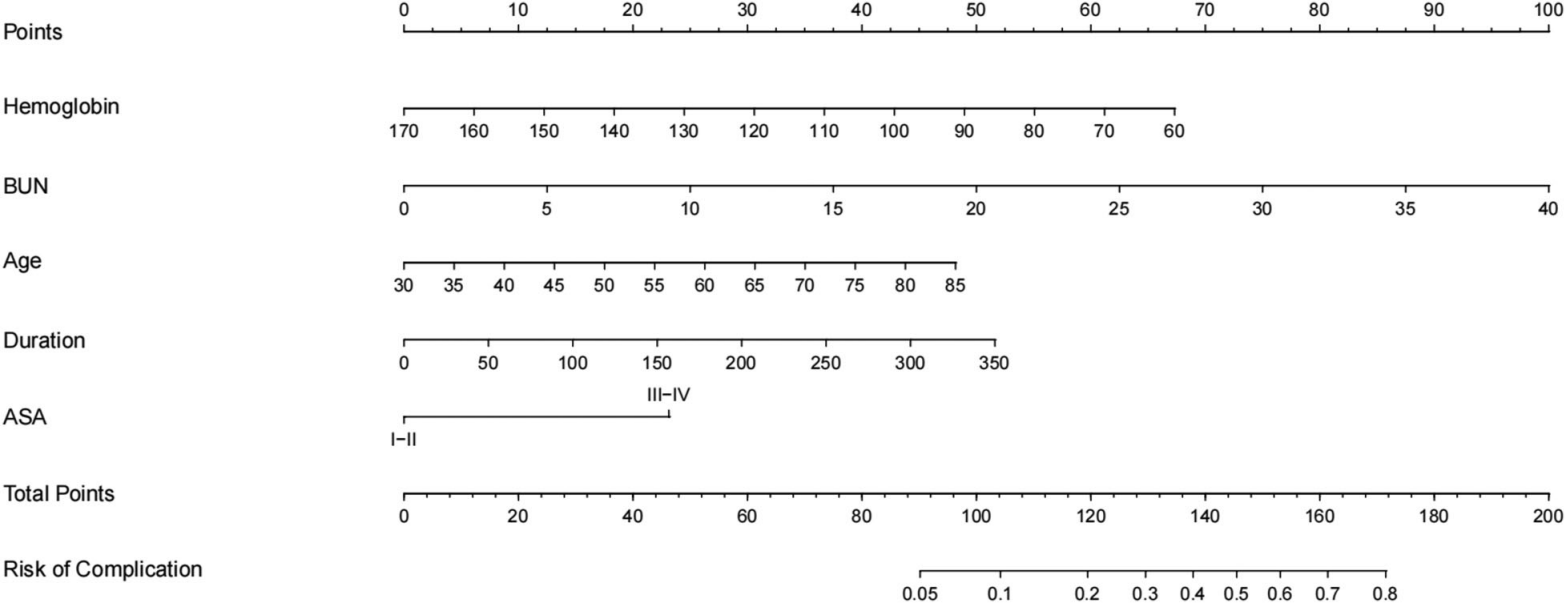
A nomogram for predicting complications within 30 days after SBTKA based on independent risk factors. BUN: Blood urea nitrogen; ASA: American Society of Anesthesiologists.

**Figure 2:**
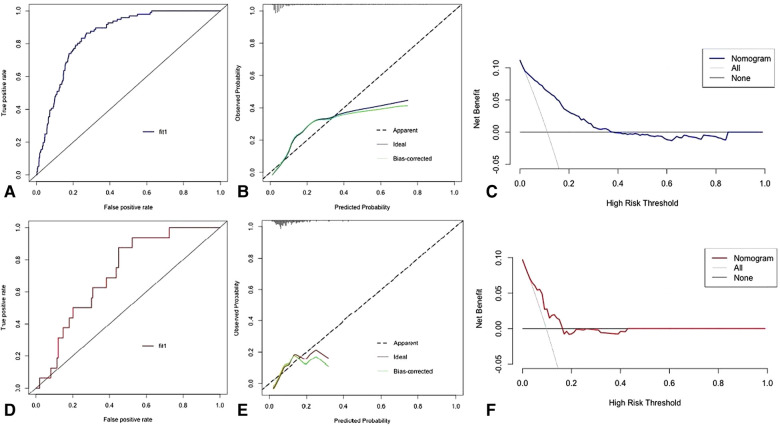
The receiver operating characteristic curve (**A**), calibration curve (**B**), and decision curve analysis (**C**) of training set. The receiver operating characteristic curve (**D**), calibration curve (**E**), and decision curve analysis (**F**) of testing set.

According to the results of multivariate Logistic regression analysis, independent risk factors predicting early complications after SBTKA were incorporated into R software to construct a nomogram model (Figure 1). The ROC of the model was plotted (Figure 2A), and the AUC was 0.851 (95% CI: 0.819–0.883), indicating that the model had a good discriminative ability. The 1,000 Bootstrap internal validation method was used to test the nomogram. Through the calibration curve, it could be seen that the nomogram predicted the risk of complications within 30 days after SBTKA and the observed probability had a good consistency (Figure 2B). The results of the DCA show that nomogram has good clinical usefulness when predicting the probability of complications within 30 days after the initial SBTKA (Figure 2C).

A total of 396 patients were included in the trial set, and 16 patients experienced complications after SBTKA. In the test set, the AUC of the nomogram was 0.818 (95% CI: 0.735–0.900) (Figure 2D), and the calibration curve was in good agreement for the prediction and observation of complications (Figure 2E). In addition, DCA showed that the use of nomogram to predict postoperative complications was net beneficial (Figure 2F).

### Comparison of the AUCs of the nomogram and a single factor for predicting complications within 30 days after SBTKA in the training and testing sets

**Figure 3:**
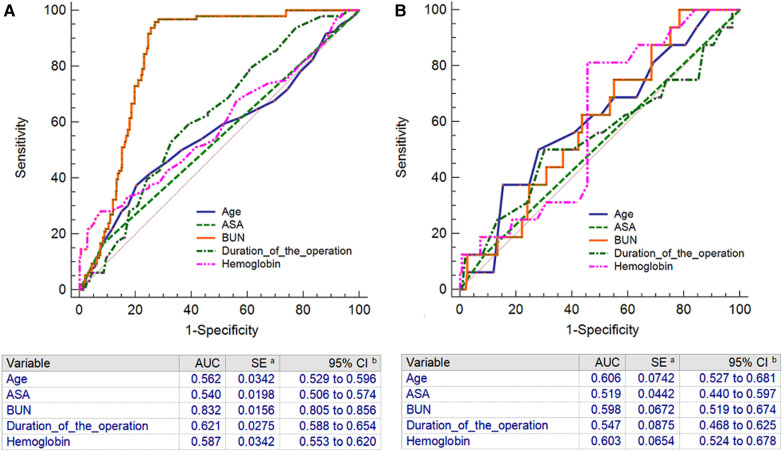
The receiver operating characteristic curves with corresponding area under the curves of nomogram and independent predictors in training set (**A**) and testing set (**B**). BUN: Blood urea nitrogen; ASA: American Society of Anesthesiologists.

In the training set, the AUC of the nomogram was significantly higher than the AUC of each independent predictor in the training set (Figure 3A). In the test set, the AUC of the nomogram was also significantly higher than the AUC of each independent predictor in the test set (Figure 3B).

## Discussion

TKA has become an effective measure for the treatment of end-stage knee diseases, which can effectively improve joint function and relieve pain (1–7, 19–22). In recent years, the nomogram is a widely used prediction tool in the field of clinical medicine, which has the ability to predict the occurrence risk of adverse events individually. This study developed a comprehensive postoperative complication prediction tool based on preoperative and intraoperative variables that accurately assessed the risk of complications within 30 days after surgery, as well as internal validation and evaluation of the model. Although some predictive models have been established in previous studies, we believe that our study improves on previous work. In the field of knee replacement, the nomogram has been used to predict the survival rate of prosthesis after knee tumor prosthesis replacement (21) and the periprosthesis infection rate (23). In addition, nomogram is used to predict the occurrence of major complications after TKA (24) and blood transfusion (25). In the above prediction tools, only patients who received unilateral TKA were included, and such studies could not be fully applicable to patients with SBTKA. On the contrary, in our study, all the subjects were patients with SBTKA.

Previous studies focused on the analysis of postoperative efficacy and perioperative complications in patients of different ages after TKA (19, 20, 26). Regardless of age, the satisfaction rate of knee surgery is more than 85%, and advanced age does not affect the efficacy of TKA (19). Other scholars have found that 70 years old is the best age for TKA (20). There was no difference in the incidence of complications among patients younger than 75 years of age who received unilateral TKA or staged bilateral TKA (14).

We collected a large number of candidate predictors, and for the screening of predictive indicators, this study comprehensively considered the statistical significance and professional significance. In the risk factor analysis, older age, lower preoperative hemoglobin levels, higher preoperative BUN levels, longer operation time, and ASA grade ≥ III were significantly associated with the incidence of complications within 30 days after surgery. These indicators were included in the prediction model of the nomogram. More importantly, four of these indicators were identifiable preoperatively, and their association with post-TKA complications has been widely reported in previous studies. Therefore, clinicians can accurately predict the risk of complications within 30 days after SBTKA preoperatively, which is crucial for early management.

The effect of advanced age on post-TKA complications has also been confirmed in previous studies (11–15). The complication rate after TKA increases with age (27–29). According to the survey, the hospital stay of elderly patients after TKA increased by 0.6–3.1 days, and the perioperative mortality rate ranged from 1.09% to 1.54% (27). Recently, however, different findings have been reported that in studies evaluating the effect of comorbidities and age on the incidence of postoperative complications after TKA, comorbidities themselves, rather than age, are the cause of increased postoperative morbidity (30). In addition, we found that preoperative lower hemoglobin was an independent risk factor for complications in patients with SBTKA within 30 days after surgery. Interestingly, a recent study found that lower preoperative hemoglobin levels were identified as an independent predictor of blood transfusion in patients after TKA (26). In this study, severe postoperative anemia was the most common complication, which well explained the influence of preoperative hemoglobin level on postoperative complications.

The results of this study suggest that longer operative time is an independent risk factor for complications within 30 days after SBTKA. Prolonged operative time can lead to increased intraoperative bleeding. In addition, prolonged exposure to the air in the operative area increases the risk of infection. At the same time, prolonged use of tourniquets increases the risk of vascular and nerve damage. It has been proved that prolonged operation time increases the incidence of postoperative complications in patients with TKA (31, 32). It has been reported in the literature that the risk of requiring blood transfusion and hospital readmission increased by 9% and 5% for each 15 min increase in the duration of TKA (33). In percutaneous kyphoplasty, operative time is an independent risk factor for hidden blood loss (34), and the findings of the above studies are consistent with the results of this study. It has been reported that the American Society of Anesthesiologists (ASA) classification has a good correlation with the incidence of complications after TKA surgery (35–38). This study also found that ASA grade ≥ III was an independent risk factor for complications within 30 days after SBTKA. The findings were consistent with previous literature that increased risk of complications after surgery was associated with an ASA score of 3 or 4 (39, 40). Interestingly, in a rigorous statistical analysis, the results showed that higher preoperative BUN levels were a risk factor for complications within 30 days after SBTKA, a finding that has rarely been reported in previous studies. The underlying mechanism between BUN levels and complications remains unclear. A possible explanation for this phenomenon is that kidney is an important organ for maintaining water, electrolytes and acid-base balance in the body, and renal insufficiency and dialysis are risk factors for infection and revision after artificial joint replacement (41, 42).

The prediction model established in this study has certain potential in clinical application, because the model has good discriminative ability and calibration degree, and the indicators included in the prediction model can be easily obtained through clinical examination at the early stage of admission, and the acquisition cost is relatively low. In this study, for the convenience of clinical application, a nomogram was developed based on the constructed prediction model, through which clinicians could quickly predict the occurrence of complications within 30 days of SBTKA and provide reference for identifying high-risk patients.

This study has some limitations. First, this study was a retrospective study, and the number of cases included was small. However, we have some clinical value by strictly validating the model through two centers. In addition, in this study, transfusion of ≥4 U red blood cells was used as one of the complications after SBTKA, and there were differences in transfusion between countries and regions. Further prospective studies are therefore needed to further validate our nomogram.

## Conclusion

Lower preoperative hemoglobin levels, higher preoperative BUN levels, longer operation time, and ASA grade ≥ III were independent risk factors for complications within 30 days after SBTKA. At the same time, a nomogram based on the above five risk factors was also developed and verified in the study.

## Data Availability

The raw data supporting the conclusions of this article will be made available by the authors, without undue reservation.
